# Firm Growth as a Driver of Sustainable Product Innovation: Mediation and Moderation Analysis. Evidence from Manufacturing Firms

**DOI:** 10.3390/ijerph18052588

**Published:** 2021-03-05

**Authors:** Ana Labella-Fernández, M. Mar Serrano-Arcos, Belén Payán-Sánchez

**Affiliations:** Department of Business and Economics, University of Almería, La Cañada de San Urbano, 04120 Almería, Spain; alf142@ual.es (A.L.-F.); marserrano@ual.es (M.M.S.-A.)

**Keywords:** firm growth, sustainable product innovation, environmental practices, barriers, labor conditions, manufacturing firms

## Abstract

Facing worldwide environmental and social concerns, manufacturing firms are trying to adopt effective environmentally friendly actions to mitigate their environmental impacts. Although the existent literature has provided many insights about the drivers of sustainable product innovation, little is known about the impact of firm growth. Thus, we intend to contribute to this gap in the literature by examining the impact that firm growth can have on sustainable product innovation. To achieve this goal, the partial least square (PLS) modeling technique was used to analyze a dataset of 3250 manufacturing firms from 39 different countries. The results reveal that firm growth exerts a positive effect on sustainable product innovation and that the relationship is partially mediated by the adoption of environmental practices. The findings also indicate that managerial barriers lessen the effectiveness of the adoption of environmental practices in facilitating the development of sustainable product innovation, while improving labor conditions increases it. However, operational barriers do not exert a significant moderating effect between the adoption of environmental practices and sustainable product innovation. These results prompt interesting insights related to theory development in environmental management and sustainable product innovation research.

## 1. Introduction

With the adoption of the 2030 Agenda, the United Nations called for action and presented 17 Sustainable Development Goals (SDGs) with the objective of joining efforts to address major global challenges. This agenda is committed to achieving sustainable development in its three dimensions: social inclusion, environmental protection, and economic development. The challenge to respond to these environmental and social concerns has dramatically changed the way many businesses operate, and these concerns have gained considerable attention in industrial activities [1].

Although the manufacturing sector provides substantial opportunities for economic growth in developing countries, this sector is a source of various forms of environmental pollution and environmental degradation [2]. Therefore, there is a growing need for adopting effective environmentally friendly practices that can mitigate the environmental impacts of this crucial sector. In fact, in the absence of positive environmental initiatives, manufacturing activities will lead to the exploitation of natural resources, the formation of huge amounts of waste, and the excessive consumption of energy [3]. Despite this, the company’s environmental performance and disclosure become increasingly significant aspects in its competitive success [4]. However, the return on investment for the adoption of environmental practices has become one of the biggest challenges for manufacturing firms [5]. Therefore, understanding how environmental practices and other relevant factors (barriers, working conditions, etc.) influence manufacturing performance is critical.

In response to these concerns, recent reviews have revealed an increasing interest in sustainable product innovation research in the last decade [6,7,8]. An extensive body of research has focused on studying the most prominent outcomes of this phenomenon: environmental, market, financial, economic, and employee performance [9,10,11,12] and competitive advantages [13,14]. Moreover, research efforts have been made to examine the factors that drive the adoption of sustainable product innovation (see recent reviews [15,16]). External drivers include market factors [17], regulatory pressures [18], and external stakeholder pressures [19], while internal drivers include organizational capabilities [20], technological capabilities [21], and environmental leadership [19], among others.

However, as noted by Tariq et al. [6], extensive research supports the notion that sustainable product innovation generally drives financial performance, but little efforts have been made to study financial performance as an antecedent of sustainable product innovation. For example, we found that Rabadán et al. [22] studied the influence of financial resources and profit levels in the adoption of eco-innovation in the agri-food industry. Similarly, Azari et al. [23] demonstrated positive and significant associations between the firm’s growth ambition and the pursuance of product and business model innovations in manufacturing and service sectors.

Moreover, although most sustainable innovation research focuses on firm financial performance and its measures, growing attention is being paid to firm growth. Recent research has empirically found that eco-innovation is positively related to firm growth [24,25]. Costa [26] showed that environmental regulations and environmental taxes enhance eco-innovation, but public grants are only appropriate in the case of eco-innovations with external benefits. At this point, eco-innovation needs to be addressed by policy actions in a structured way, as it is considered a driver of the environmental and innovative performance, generating more sustainable business models and competitive advantages [26]. However, the effect of firm growth on sustainable product innovation has not received empirical attention. Consequently, with this study, we aim to contribute to address this gap in the literature, considering firm growth as an antecedent for the implementation or higher achievement of sustainable product innovation within firms.

According to the resource-based view [27], previous research theorizes that organizations first acquire resources to build superior capabilities, change organizational practice, and then achieve superior outcomes (e.g., sustainable product innovation) [28,29]. In this way, it could be interesting to study the indirect mechanisms that change organizational practice and improve sustainable product innovation outcomes. The adoption of environmental practices is regarded as best practice in regard to the aim of minimizing the impact of a firm on the natural environment [30]. The adoption of these practices exhibits the extent to which environmental concerns are integrated into businesses, as environmental management is commonly operationalized through environmental initiatives or practices [31]. Although the adoption of environmental practices often requires high initial costs [32], firms that experience growth have more resources, which, in turn, influence the adoption of environmental practices. Thus, this study also aims to propose the adoption of environmental practices as a transformation mechanism through which firm growth influences sustainable product innovation.

In adopting environmental practices, many firms frequently face barriers, such as the lack of financial resources and managerial commitment, that hinder the effectiveness of the environmental actions [33]. Operational and managerial barriers play a critical role when firms address environmental management [34,35]. Therefore, our objective is also to further explore the role of operational and managerial barriers in moderating the link between the adoption of environmental practices and sustainable product innovation.

In addition, in pursuing sustainable development, organizations are not only paying attention to their impact on the environment but also on society. Organizations are increasingly managing social issues at work, including improving labor conditions, guaranteeing human rights, health and safety, gender equality, and the inclusion of disabled and marginalized people [36]. Labor conditions could represent a key mechanism in environmental management. When organizations create a positive work environment for the workforce, employees will be more likely to engage in environmental initiatives of organizations [37]. Thus, it would also be interesting to investigate the moderating role of improving labor conditions on the relationship between the adoption of environmental issues and sustainable product innovation.

To achieve these goals, we used data gathered from Flash Eurobarometer No. 486 [38]. This cross-national sample comprises 3250 manufacturing firms from 39 European countries. We assess our proposal model using structural equation modeling.

This research makes several contributions to the existing literature. First, this paper examines the relationship between firm growth and sustainable product innovation, thereby contributing to the literature on sustainable product innovation by extending its potential antecedents. Second, our insights enhance the understanding of the mechanisms by which firm growth affects sustainable product innovation by studying the mediation effect of the adoption of environmental practices. Finally, we further explore the indirect effects of the adoption of environmental practices on sustainable product innovation by exploring the moderating roles of operational and managerial barriers, as well as the improvement in labor conditions. By doing so, this study contributes to the literature on environmental management by shedding light on the factors that influence the effectiveness of environmental management, and it also contributes to the understanding of managing social issues in environmental management.

The remainder of this paper is organized as follows: the next section presents the theoretical model and proposes the five hypotheses; Section 3 describes the methodology and presents the analysis and results; the last section of the paper discusses theoretical contributions, managerial implications, and the limitations and presents the guidelines for future research.

## 2. Literature Review and Hypotheses

Considering the increasingly competitive context of the contemporary economy providing the possibility of offering products in many market segments, firms find the need to differentiate from competitors by generating sustainable competitive advantages, which are highly supported by innovation processes [15]. Nowadays, given the environmentally related growth limits organizations face, innovation is seen as a latent need to be adopted taking into consideration social and environmental concerns, contemplating organizational growth through low environmental impact practices, such as sustainable innovation practices [8,15,39,40].

Concretely, sustainable product innovation successfully combines the exploitation of new knowledge and sustainability, bringing new products or technologies to the market while having minimal possible impact on the environment [41,42,43]. Varadarajan [44] (p. 4) defined this process as “a firm’s introduction of a new product or modification of an existing product whose environmental impact during the lifecycle of the product, spanning resource extraction, production, distribution, use, and post-use disposal, is significantly lower than existing products for which it is a substitute”. Accordingly, sustainable product innovation includes products’ technical design, R&D activities, and manufacturing and management functions, as well as the commercial activities needed to market a new (or improved) product [45], all of which need to be achieved and supported within the firm. Although this field of research has been expanding rapidly in recent years [16], we still do not know very much about it [46].

Many studies have been conducted to detect the drivers and motivations for companies to adopt sustainable product innovation [6,15,16]. Some of them are market-oriented, including demand characteristics, stakeholders’ behavior, and governmental regulations [46,47,48,49,50], while others focus on interfunctional collaborations with technology dissemination, vertical communication [46,51,52,53], and internal organizational decisions and management variables, with firm culture and high levels of internal and external integration representing key factors [15,45,54,55,56,57]. Additionally, many scholars have paid attention to sustainable product innovation as a driver to improve organization performance and competitiveness and business success, which ultimately promote firm growth (in terms of increased income, for instance). However, very few studies have focused on the relationship between the growth of a firm and the development of sustainable product innovation practices, which has been studied (if so) as a consequence of the implementation of these practices and not as a determinant factor for it [6,24,58]. In fact, economic performance, success, and firm growth are variables that have mostly been analyzed as a result of the relation rather than the driver, and above all, regarding environmentally related innovation practices [16,46,58,59,60,61,62].

### 2.1. Firm Growth and Sustainable Product Innovation

Some attempts have been made to analyze the effects of certain firm characteristics, which could resemble various kinds of internal and external growth, on sustainable product innovation. For instance, Muñoz-Pascual et al. [45] analyzed a firm’s external growth in the sense of accessing new markets through internationalization strategies as a positive driving force for sustainable product innovation. In this situation, firms face increasing competition to the extent that it stimulates their efforts to reduce costs, increase product quality, and gain in flexibility. This forces them to continuously invest in technology and update their products [45], adapting them to the new markets and social requirements. As a consequence of this growth, organizations increase their return and have a better access to economies of scale, important factors given the considerable fixed costs involved in product innovation [63] and the resource requirements that sustainability issues entail.

Other examples regarding internal factors can be found in the works of Leonidou et al. [64] and Rehfeld et al. [65]. On the one hand, both studies connect firm size with green product innovation, highlighting the positive impact of the size on the firm’s green innovation practices [16]. On the other hand, Leonidou et al. [64] asserted that the availability of slack resources in a firm positively affects the development of sustainable product innovation. Slack resources are well known in the management literature as the excess obtained from a firm’s financial resources over those resources needed to maintain its operations. Thus, slack resources serve as a safeguard from short-term performance needs, allowing managers to propose new strategies and a longer-term plan for the company [64]. Given the significant short-term expenses that environmental investments require, the presence of slack resources makes firms better able to make such investments [64,66].

As previously mentioned, the innovation process involves several phases, from discovery to implementation [67], with success outcomes depending on the efforts that a firm can make. Innovation is costly in general, making resources and efforts for this process an important player in innovation success [68] and, thus, relying on the resources that a firm has at their disposal in such practices. This implies that the bigger the firm, the more successful the sustainable product innovation, given the greater efforts and resources available to this end.

Technological progress is one of the most important forces enabling sustainable development [69,70] and, by extension, sustainable product innovation [15]. Several scholars have highlighted the great importance of investing in tools and methods by developing new technologies and qualifying their productive systems, as well as new product development processes, to be able to embrace environmental sustainability and succeed in sustainable product innovation [54,71]. Montalvo [72] remarked on the important influence that technological capabilities (such as qualified HR, equipment, and laboratories) and institutional capabilities have on the implementation of greener technologies in a firm [15]. In the same vein, Horbach et al. [18] highlighted resource availability and technological competences as important internal factors that promote environmentally sustainable product innovation, as they give firms the capabilities needed to find a way to respond to external environment requirements and inputs more efficiently

Concretely, sustainable product innovation requires financial, human, and time resources to be developed, and they will be more easily available if a firm has grown in terms of revenue generation and increased in workforce. Additionally, prior studies following the resource-based view theorize that organizations first acquire resources to build superior capabilities, deploying these resources and capabilities afterwards to change organizational practice, thereby finally achieving superior performance outcomes (e.g., sustainable product innovation) [27,28,29].

To the end, we think that high levels of firm growth may reflect a greater chance of investing in sustainable product innovation. Consequently, we posit the following hypothesis:

**Hypothesis 1 (H1).**  *Firm growth is positively associated with sustainable product innovation.*

### 2.2. The Mediating Role of the Adoption of Environmental Practices

The recent worldwide reinforcement of environmental regulations (as well as the need for compliance with them) has motivated firms to adopt environmental practices as part of their daily activities [73]. Environmental practices are regarded as the best practices aimed at minimizing the impact of the firm on the natural environment [30]. Thus, their adoption exhibits the extent to which environmental concerns are integrated into environmental management in businesses, which is commonly operationalized through environmental initiatives or practices [31].

Some of the environmental practices that firms can adopt relate, for instance, to the analysis of a product life cycle or eco-design, which is becoming a global trend above all in the engineering, architecture, and design fields of expertise [62]. With these practices, organizations gain ideas for the development of new products, systems, and services and, at the same time, the optimization of the production process while minimizing their environmental impact through the use of non-renewable resources [62]. Other highly used and adopted environmental practices are cleaner production methodologies, the reuse or recycling of materials, energy saving efforts and the use of sustainable energy sources, and reductions in the consumption of natural resources (e.g., saving water), which help firms to reduce their waste and emissions [46].

Several scholars have made efforts to identify the key drivers of these practices [74,75], also focusing on the manufacturing sector [76,77], and they highlighted the key role of industry and the cultural environment on the assimilation of environmentally friendly behaviors [1]. Concretely, the most important and effective drivers identified in the literature regarding the adoption of environmental practices in firms are related to regulations, customer pressures (external), top management support, employee commitment, and cost savings [1].

Intense debate has been held in the literature regarding the relationship between environmental practice adoption and financial performance [78,79,80], with research being mostly focused on the impact that the adoption of environmentally friendly actions has on a firm’s performance [81,82] despite the uncertainties of the bidirectional relationship, which “have been called into question” [1] (p. 692). More concretely, in manufacturing industries, the literature expands on this debate, showing mainly positive results in the relation [61,83,84].

However, in regard to this debate, we think that a firm’s performance and growth can be drivers of environmental practice adoption. As previously discussed, firm growth via revenue generation and increases in workforce, for example, provides available resources (financial, human, and time resources), access to new resources and knowledge [85], and the development of the capabilities needed to implement environment-related practices within a firm. Muñoz-Pascual et al. [45] highlighted the main factors leading to the adoption of environmental practices in terms of exports, investments in human resources, organizational learning capabilities, and knowledge sharing. We see all these elements related to firm growth in the sense that the more the firm increases in size, the more successfully these practices are achieved and the greater the effects they have on the company in terms of environmental concerns.

As Miras-Rodríguez et al. [1] stated, there is no doubt about the major investments that the adoption of environmentally friendly actions requires in a firm in terms of training and equipment improvements and acquisitions. Additionally, these costs are easily recognizable, but this is not true for the benefits given the time lags they usually entail [86]. Therefore, it is widely accepted in the literature that the adoption of environmental practices frequently entails high initial costs [1,32], and firms that experience growth have more resources to invest, which, in turn, influence the adoption of environmental practices.

On the other hand, it has been proven in the literature that the adoption of environmental practices has a direct effect on sustainable product innovation [15,45,46], creating an immediate and visible improvement in organizational efficiency and firms’ product innovation processes [87]. In this sense, Muñoz-Pascual et al. [45] concluded that the adoption of environmental practices is positively related to product innovation, which leads to this becoming sustainable product innovation. This may be due to the need for applying more efficient consumption methods and waste recycling during sustainable product innovation processes, reducing a firm’s operational costs afterwards [88]. Following this, the literature further argues that achieving a proactive environmental strategy needs changes in firm routines and operational methods promoted by environmental practices [57], and that the adoption of these practices also assists in reaching better safety standards and healthier working conditions [89]. Chen [55,56] showed the need for developing a set of green competences that influence management processes to achieve superior sustainable product performance. More concretely, Hemel and Cramer [54] listed the main solutions that most companies use to develop environmental innovation: investing in material recycling and energy consumption, using recycled materials, and extending product lifespans.

Having established the connections between firm growth and the adoption of environmental practices and between the adoption of these practices and sustainable product innovation, we now consider the question of how firm growth influences sustainable product innovation by integrating environmental practices within the management and operations of the firm. Central to our argument is the idea that those firms that display sustainable product innovation will not necessarily be those that simply grow but, rather, it will be those that successfully adopt environmental practices to support firm growth and finally reach sustainable product innovation. In this sense, the adoption of environmental practices, by providing the tools to minimize a firm’s impact on the natural environment [30], is responsible for translating the financial, technical, human, and time resources obtained through firm growth into sustainable product innovation success.

On the basis of the above, it seems reasonable that sustainable product innovation is strongly influenced by the extent and intensity of the adoption of environmental practices, which, in turn, are dependent on several resources and knowledge to be invested in the short term that are available with the help of firm growth. Hence, we argue that firm growth can influence and improve sustainable product innovation by encouraging the use of and, ultimately, the adoption of environmental practices, leading to the following hypothesis:

**Hypothesis 2 (H2).**  *The relation between firm growth and sustainable product innovation is positively and partially mediated by the adoption of environmental practices.*

### 2.3. The Moderating Role of Managerial Barriers

Managerial barriers reflect the existing insufficiency in the procedures that inhibit the environmental operation of a company and the adoption of strategies [90,91]. According to Dubey et al. [92] and Zhang et al. [34], when managerial barriers are high, they can lead to a lack of environmental commitment, such as poor commitment between functions, an inefficient organizational culture for environmental management, and the reduction in the probability of a shared vision of problems between departments. Therefore, the environmental strategy may be less useful in facilitating environmental management actions [92]. Additionally, a high level of managerial barriers can lead to a poor resource base and inefficient environmental concern [93]. In turn, a proactive environmental strategy may not be successful as a consequence of an organization with different interpretations [94].

In general terms, a sustainable and innovative organization simultaneously seeks to succeed in economic terms while not affecting the availability of resources in its ecosystem and respecting the capacity to support the environment [62]. Hence, sustainable product innovation arises as an opportunity to launch “a new product on the market that meets the pressures brought about by the legislation and the global society” [62] (p. 88). Accordingly, given the complex nature of sustainable product innovation, cross-functional coordination, integration, top management support, and resource investment are substantial for the implementation of it [95,96]. In this sense, a high level of managerial barriers can widen the relationship between the adoption of environmental practices and the innovation of sustainable products [97].

On the contrary, when the level of managerial barriers is low, the adoption of environmental practices is more likely to invest in innovation of sustainable products [96,98]. In addition, an environmentally friendly organizational culture can enable firms to reduce the gap between its environmental strategies and its real environmental practices [62].

Following this reasoning, it is expected that high managerial barriers will undermine the relationship between the adoption of environmental practices and sustainable product innovation. Therefore, we propose the following hypothesis:

**Hypothesis 3 (H3).**  *Managerial barriers negatively moderate the relationship between the adoption of environmental practices and sustainable product innovation.*

### 2.4. The Moderating Role of Operational Barriers

Operational barriers encompass a series of obstacles in operations management systems, such as poor technical support, high costs, and/or inadequate measurement metrics to identify and address environmental issues [35,99]. Drawing upon contingency theory, the success of the proactive environmental strategy depends on the context [94,98]. Previous works have shown that when the operational barriers are high, they can hinder the implementation of green operational practices as well as reducing the effectiveness of green human resource management and environmental legitimacy in facilitating the implementation of environmental practices [100,101]. In this sense, Bhanot et al. [93] highlighted that a high level of operational barriers may cause an environment-oriented association to avoid investing in environmental practices/initiatives according to the low expected return on investment. Additionally, it is crucial to consider (limited) resources which can reduce the effectiveness of committed capabilities and important resources, and, therefore, they need to be divided to address technical hurdles [102]. At the same time, when operating barriers are high, they can weaken the contribution of environmental management (e.g., green human resource management) to the implementation of green operational practices due to the firm avoiding conducting green operational practices or the low probability of their successful implementation [62].

According to our research context, little is known about the negative effect of operational barriers in the process of adopting environmental practices and innovation of sustainable products. Accordingly, the expected low availability of resource commitment and high uncertainty of environmental practices in the context of high operational barriers can detract from the successful implementation of sustainable product innovation by companies. On the contrary, a low level of operational barriers can make firms better at identifying underperforming key sustainable operational problems and taking full advantage of the commitment of resources to efficiently solve environmental issues [34].

In this context, the adoption of environmental practices can contribute to the implementation of sustainable product innovation more efficiently. Accordingly, it is expected that high operational barriers will undermine the relationship between the adoption of environmental practices and sustainable product innovation.

Thus, we propose the following hypothesis:

**Hypothesis 4 (H4).**  *Operational barriers negatively moderate the relationship between the adoption of environmental practices and sustainable product innovation.*

### 2.5. The Moderating Role of Labor Conditions

Social performance refers mainly to indicators such as improvement in the safety and health of employees, better working conditions, and fair treatment of workers in terms of equity and diversity [36]. Consequently, this management makes employees more motivated and more environmentally aware and, as a consequence, generates greater innovation [45]. For many organizations, providing an enjoyable and healthy workplace for employees has become essential. Previous works have demonstrated that the well-being of workers and their labor conditions influence the attitudes, perceptions, and behaviors of employees and, consequently, the success of the organization [103]. This is the reason why employees with greater well-being perform their work productively, which benefits the company as employees engage and display pro-environmental behavior in the workplace [104]. Therefore, firms around the world are increasingly encouraging their employees to engage in voluntary pro-environmental behavior to enhance environmental performance [105].

Previous work has shown that when companies create a favorable work environment for employees, the employees are more likely to engage in the green initiatives of the organization [37]. In this sense, the establishment and achievement of various pro-environmental initiatives employed at the company level depend on the pro-environmental behaviors of employees [106]. According to Andersson et al. [107], employee green behavior refers to any evaluable behavior of an individual that contributes to achieving environmental sustainability in the workplace. According to De Roeck and Farooq [108], this behavior is associated with environmentally friendly behavior that employees perform in a company, such as reusing materials and maintaining sustainable policies, among others. For example, in the study conducted by Ahmed et al. [37], the pro-environmental behavior that employees display in the workplace in regard to the protection of the natural environment includes printing on both sides of paper, turning off the lights, cleaning the working environment, and using stairs instead of elevators. The adoption of environmental practices assists in the achievement of better safety standards and healthier labor conditions [89].

As such, it is expected that positive labor conditions will strengthen the relationship between the adoption of environmental practices and sustainable product innovation. Based on this argument, we propose the following hypothesis:

**Hypothesis 5 (H5).**  *The improvement in labor conditions positively moderates the relationship between the adoption of environmental practices and sustainable product innovation.*

**Figure 1:**
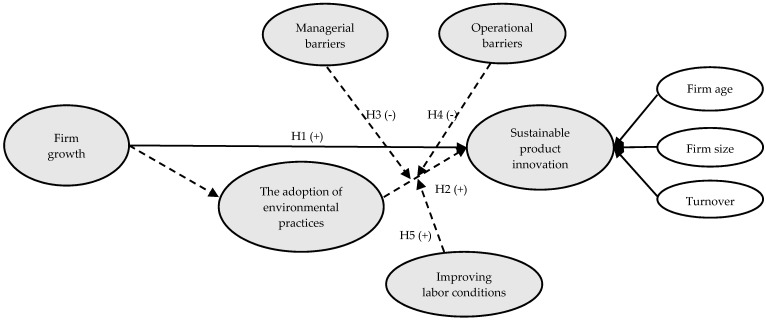
Conceptual model. **Note**: + positive relationship, - negative relationship.

The conceptual model for this research and the formulated hypotheses are presented in Figure 1.

## 3. Data and Methodology

### 3.1. Sample

Eurobarometer surveys are conducted on behalf of the European Commission and examine public opinion and behavior on many different topics. Data were gathered from the “Flash Eurobarometer No. 486: SMEs, start-ups, scale-ups and entrepreneurship” survey [38]. This specific survey covers interesting topics, such as sustainability, innovation, and digital technologies. Access to this cross-national database was provided by the GESIS. Use of the Eurobarometer surveys is relatively common in environmental management empirical research (e.g., [109,110]).

The fieldwork was conducted between February and May 2020. Interviews were conducted by phone in their respective national language. The sample data in the study consist of 16,365 companies and cover many sectors from 39 countries. These countries are 27 European countries and 12 non-EU countries to have a comprehensive view of different continents around the world.

As this study only focuses on manufacturing firms, we discarded all cases of companies that do not belong to the manufacturing sector (13,115 cases). Therefore, the used sample is composed of 3250 firms from the manufacturing sector. Table 1 shows the main characteristics of the sample. The sample is clearly dominated by small and medium-sized entreprises (SMEs). Two fifths of the sample is composed of microenterprises with fewer than 10 members of staff (40.1%), and there are 906 firms with between 10 and 49 employees and 745 firms with between 50 and 249 employees. Moreover, EU countries represent 76.36%, while non-EU countries represent 26.64% of our sample.

### 3.2. Measures

Table A1 in Appendix A shows the detailed questionnaire items for each one of the variables of the study.

#### 3.2.1. Dependent Variables

To measure sustainable product innovation, we used two items extracted from the Eurobarometer survey: (1) the introduction of innovation with environmental benefits, including innovations with an energy or resource efficiency benefit during the past 12 months, and (2) if the firm is actively developing sustainable products. Prior empirical studies have also used dichotomous variables as measures of innovation outcome (e.g., [111]).

#### 3.2.2. Independent Variables

Firm growth was operationalized in terms of employees and turnover growth since 2016. These two variables were recodified in dichotomous variables (1 = if the firm has grown by less than 30% or by at least 30% and 0 = if the firm has decreased or remained stable). Therefore, after the recodification process, these variables (employees and turnover growth) result in dichotomous variables (1= firm growth and 0 = no firm growth/firm decline).

The adoption of environmental practices was measured by practices that firms are actively adopting. The scale includes three dichotomous variables: (1) recycling or reusing materials, (2) reducing consumption of or impact on natural resources (e.g., saving water or switching to sustainable resources), and (3) saving energy or switching to sustainable energy sources. Previous research has used similar environmental practices to operationalize this construct (e.g., [45,112]).

Operational and managerial barriers were measured by two dichotomous variables covering whether the company faces any of these barriers to innovation. Managerial barriers consist of the following variables: (1) lack of skills, including managerial skills, and (2) lack of willingness among management staff. Operational barriers were operationalized through these variables: (1) lack of technology infrastructure, and lack of financial resources, including those from available support schemes. These two dimensions are composed of similar variables to those in previous studies (e.g., [34]).

Finally, the improvement in labor conditions was operationalized through two dichotomous variables: improving the working conditions of employees and promoting and improving diversity and equality in the workplace.

#### 3.2.3. Control Variables

To account for possible alternative explanations, we included a set of variables at the organizational level of analysis. In accordance with previous research, we used control variables regarding organizational characteristics and corporate finance [113]. Firm age was operationalized by the duration from the year in which the firm was established to the sample year (2019). Firm size was measured by the number of employees. Finally, the annual turnover was measured categorically, represented by eight categories: 150,000 or less; more than 150,000 and up to 760,000; more than 760,000 and up to 1.5 million; more than 1.5 million and up to 3 million; more than 3 million and up to 7.6 million; more than 7.6 million and up to 15 million; more than 15 million and up to 76 million; and more than 76 million.

#### 3.2.4. Common Method Bias

Data collection from different sources is the most ideal research method in behavioral sciences to avoid common method bias (CMB). However, the anonymity policy of the European Commission did not allow us crossing data from the database used in this study with data from other sources to assuage CMB [114]. We therefore applied statistical methods as CMB might potentially be present in the dataset since all the dependent and independent variables in this study were collected at the same time from the same respondent. First, we used Harman’s single-factor method [114] as it was commonly used in previous research (e.g., [115]). All survey items were included in the model to assess if most of the variance was accounted for by one general factor. We carried out a principal component analysis with the unrotated solutions. The results show that, in addition to extracting five factors based on eigenvalues over 1, the variance explained by the general factor was 19.9. This result indicates that CMB is not a major concern in the study.

Second, following the recommendations of Kock [116], CMB can be addressed by analyzing the full collinearity of the PLS model. In this analysis, all the variables will be regressed against a common variable and the variance inflation factor (VIF) values must not exceed the threshold of 3.3; if this result is attained, then no bias from the single source data is present. The obtained VIF values fulfill the threshold criteria. Thus, both approaches indicate that CMB is not a serious concern in this study.

### 3.3. Analysis

We used structural equation modeling (SEM) to test the proposed hypotheses. SmartPLS 3.2.8 was applied to conduct statistical analysis. Partial least square PLS-SEM is a multivariate, non-parametric technique employed for estimating path models with latent variables [117]. According to Hair et al. [117], several reasons can be put forward for this choice. First, PLS path modeling is acknowledged as a suitable analytical technique for causal-predictive analysis. Second, PLS-SEM is an appropriate method for examining complex research frameworks, particularly for research models involving mediation and moderation. Third, this technique works with any type of variable (ordinal, categorical, or dichotomous variables) [118]. Finally, PLS-SEM has been widely employed in previous innovation research (e.g., [119]).

Two stages of evaluation of PLS-SEM modeling are carried out [117]. In the first stage, an evaluation of the measurement model is investigated for validity and reliability. In the second stage, an evaluation of the structural model is carried out for hypothesis testing.

### 3.4. Measurement Model Evaluation

For the measurement model, composite reliability (CR), the loadings, and average variance extracted (AVE) were assessed. Table 2 shows the results. The values for CR were also well above the 0.70 value, which indicates good internal consistency between items in a construct. The loadings were acceptable, with only two loadings being less than 0.732 [120]. Moreover, AVE values were above the recommended threshold of 0.50. Both loadings and AVE values adequately demonstrate convergent validity [121].

Next, we examined the measurement model for discriminant validity, and for this, we used heterotrait–monotrait (HTMT) criteria. HTMT analysis has proven to be superior among the methods for assessing discriminant validity [122]. Table 3 indicates that HTMT ratios were below the threshold value of 0.85. Hence, discriminant validity is also accomplished.

### 3.5. Structural Model Evaluation

After processing the measurement model, the structural model must be estimated. This process was conducted through the bootstrapping approach, with a 95% significance interval and 5000 subsamples, as recommended by Hair et al. [117].

For this procedure, we included the coefficient of determination (R^2^), predictive relevance (Q^2^), and the variance inflation factor (VIF). Table 4 shows the results of the assessment of the structural model. First, R^2^ evaluates the quality of the adjusted model. The condition for the dependent variables’ R^2^ values is that they should be greater than or equal to 0.10 [118]. As shown in Table 4, the values of R^2^ are above the recommended value and indicate a slightly moderate level of predictive accuracy [117]. Next, Stone–Geisser’s predictive relevance (Q^2^) values were calculated in order to assess if the data points of indicators in the reflective measurement model of the endogenous construct can be predicted accurately. The interpretation of the value takes 0 as a reference level, and the model has a predictive value when the indicator is positive. The Q^2^ values were greater than zero, indicating strong predictive power. Finally, the traditional variance inflation factor (VIF) evaluates multicollinearity problems. The VIF values were lower than the threshold value of 3.3, indicating no collinearity between the independent and dependent variables [123,124].

For hypothesis testing, the structural model included several tests, such as estimating path coefficients and their significance [117]. Table 5 shows the results for the hypothesis testing. The study findings reveal that firm growth is positively and significantly associated with sustainable product innovation (β = 0.036, *p* = 0.008, *t*-value = 2.405). Hence, we found support for Hypothesis 1 in our model.

We also established that the implementation of environmental practices partially mediated the relationship between firm growth and sustainable product innovation. To test this mediation effect, this study examined the significance of the direct and indirect effects by employing the bootstrapping function of Smart PLS. Hair et al. [117] suggested that if the indirect effect is significant and the direct effect is not significant, there will only be indirect mediation (full mediation), while if both indirect and direct effects are significant and point in the same direction, there will be complementary mediation (partial mediation). Table 5 shows that the indirect effect was significant (β = 0.028, *p* = 0.000, *t*-value = 4.353) as well as the direct effect. This demonstrates the partial mediation effect in our sample. Thus, Hypothesis 2 is supported.

For analysis of the moderation effects, we used a two-stage approach as recommended by Hair et al. [117] for the formative construct. We estimated a different model, introducing the interaction terms. It should be noted that when three moderators were included, there was an increase in the explained variance (R^2^) for sustainable product innovation by 5.3% (from 0.286 to 0.302) and an increase in the predictive relevance (Q^2^) of the model by 3.8% (from 0.176 to 0.183), which indicates the presence of moderating effects. We found that managerial barriers negatively and significantly moderate the relationship between the implementation of environmental practices and sustainable product innovation (β = −0.038, *p* = 0.010, *t*-value = 2.317), thereby supporting Hypothesis 3. However, we did not find support for the moderating effect of operational barriers (β = 0.001, *p* = 0.467, *t*-value = 0.083). Therefore, Hypothesis 4 was not supported in the present sample. Finally, we found that the improvement in labor conditions significantly moderates the relationship between the implementation of environmental practices and sustainable product innovation (β = 0.145, *p* = 0.000, *t*-value = 8.617). Consequently, Hypothesis 5 is supported by our data.

## 4. Discussion and Conclusions

According to our findings, from a generic perspective, this research provides empirical evidence on the role that some significant variables in sustainable development (i.e., firm growth, the adoption of environmental practices, operational and managerial barriers, and working conditions) play in explaining sustainable product innovation. In summary, this research describes the underlying mechanisms that explain the effect of firm growth on sustainable product innovation in the manufacturing sector. For a better and complete understanding of this relationship, this study also analyzes the mediation effect of the adoption of environmental practices. In addition, the indirect effects of the adoption of environmental practices on sustainable product innovation are explored in regard to the moderating roles of operational and managerial barriers as well as the improvement in labor conditions within firms.

First, this study shows that firm growth in terms of employees and turnover increase since 2016 contributes to the introduction of sustainable products. Sustainable product innovation is associated with the introduction of innovation in a firm’s product with environmental benefits, including energy or resource efficiency. Hence, these findings reflect that firm growth plays a significant role in the development of sustainable product innovation. This finding empirically clarifies that when organizations experience long-term growth, they have more resources to invest and capabilities to develop sustainable product innovation.

Second, the analyses also validated the positive mediation effect of the adoption of environmental practices (i.e., recycling or reusing materials, reducing consumption of or impact on natural resources, and saving energy or switching to sustainable energy sources) in the relationship between firm growth and sustainable product innovation. An explanation for the above finding lies in the argument inherent in resource-based view theory [27]. Once organizations have acquired resources, they can strengthen their capabilities and change organizational practices, which leads to better outcomes [28,29]. In this regard, this study confirms the importance of the adoption of environmental actions as a mechanism to change organizational practice and contribute to better sustainable outcomes within organizations.

Third, these results show that managerial and operational barriers condition sustainable product innovation in different ways. Managerial barriers (i.e., lack of skills, including managerial skills, and lack of willingness among management staff) have a negative moderating effect, since the relationship between the adoption of environmental practices and sustainable product innovation demonstrated a weaker link as a consequence of the moderating effect of high managerial barriers. However, in addition to the findings of the study conducted by Zhang et al. [34], the findings of this study reveal that operational barriers (i.e., lack of technology infrastructure and lack of financial resources, including available support schemes) do not have a moderating effect that explains the link between the adoption of environmental practices and sustainable product innovation. This might be due to the fact that the adoption of environmental practices can help organizations address operational barriers by developing necessary capabilities and enhancing sustainable resource application. Therefore, these results reflect the relevance of the managerial barriers since they decrease the impact of the adoption of environmental practices on sustainable product innovation in the manufacturing sector. On the contrary, improving labor conditions has a positive moderating effect, since the link between the adoption of environmental practices and sustainable product innovation is strengthened as a result of the moderating effect of the improvement in labor conditions (e.g., promoting and improving diversity and equality in the workplace). The results indicate that when the level of improvement in labor conditions is high, organizations that have adopted environmental practices are more likely to develop sustainable product innovation successfully. Therefore, the relevance of firms to offer and promote improvements in the safety and health of employees, better working conditions, and treatment of workers in terms of equity and diversity is noteworthy.

### 4.1. Theoretical Contributions

From a theoretical point of view, this research provides interesting insights related to theory development in the fields of environmental management and sustainable product innovation research. Sustainable innovation practices have received much attention in the literature, also centered on the study of manufacturing firms with the aim of understanding their drivers and how environment-related outcomes can be fostered [77]. Sustainable product innovation, together with its determinants and consequences, has been widely studied in the literature [6,15,46]; however, there are still some firm variables that have not been brought to the forefront and that we think are important, e.g., firm growth. Although some efforts have been made to study financial resources as an antecedent of sustainable product innovation [22], firm growth as a driver of sustainable product innovation has not received attention in the literature. Thus, this study introduced firm growth, in terms of employees and turnover increase, as a driver for sustainable product innovation, contributing to the introduction of sustainable products in the firm. Hence, our results show that firm growth plays a significant role in the development of sustainable product innovation, something that has not been paid attention to previously in the sustainable and environmental management literature. Furthermore, while it has been studied in relation to sustainable product innovation, we introduced the mediating role of the adoption of environmental practices for which we also found a gap in the literature in terms of its relation to firm growth as an antecedent. In this sense, we found a partial and positive mediation effect of the adoption of environmental practices on the relationship between firm growth and sustainable product innovation. Additionally, previous research has studied managerial and operational barriers as moderating variables in the context of proactive environmental strategy [34]; however, no specific study seems to have attempted to examine these moderating roles and the improvement in labor conditions in regard to the relationship between the adoption of environmental practices and sustainable product innovation. Therefore, this research enhances the current knowledge in the literature of environmental management by studying the moderating roles of managerial and operational barriers and labor conditions in terms of their relationship with sustainable product innovation.

### 4.2. Managerial Contributions

From the managerial perspective, this research demonstrates the relevance of firm growth and the adoption of environmental practices in the success of sustainable product innovation. Hence, organizations in the manufacturing sector should pay particular attention to the effect of firm growth on sustainable product innovation to build superior capabilities, change organizational practice, and achieve superior outcomes by implementing innovation practices. Accordingly, it is relevant for organizations in such a sector to know how to implement and enhance their sustainable product innovation. However, the findings of this research show that organizations face obstacles such as the existence of managerial barriers, which play an important role when organizations begin to adopt environmental practices with the aim of sustainable product innovation. This is the reason why it is essential to decrease managerial barriers and provide resources in order to decrease the lack of skills (including managerial skills) and some mechanisms in order to create environmental awareness, thereby ensuring that the lack of willingness among management staff does not affect the sustainable objectives of firms. In addition, the empirical evidence provided by this study supports the necessity of improving working conditions of firms’ employees in terms of promoting and enhancing diversity and equality in the workplace. For this reason, firms should be aware of the importance of providing better and healthy labor conditions during sustainable product innovation. Consequently, such organizations should invest in various indicators, such as better working conditions, fair treatment of workers, and improvement in the safety and health of employees. Furthermore, when employees see an improvement in their well-being, they perform their work in a more productive and motivated manner, which leads to them being involved and showing pro-environmental behavior in the workplace.

### 4.3. Limitations and Future Research Directions

There are some limitations to this research that require further consideration. First, this study was based on firms from the manufacturing sector; hence, a replication of this study in other sectors (such as the service sector) could be employed to compare the findings and to study if there are specific differences between sectors. Second, the studies that explore sustainable product innovation and firm growth as one of its antecedents have received little attention. Third, according to the moderating effects, only three variables were measured; therefore, further research should be conducted on other potential moderating effects, for example, novelty-centered business model design and efficiency-centered business model design (e.g., [125]). Firm growth could also be studied as a possible moderator of the relationship between sustainable product innovation and its widely studied drivers, such as cleaner production [62], corporate social responsibility [126], and innovation drivers (e.g., quality of products/services, productive capacity, flexibility in production/service, and reduction in operating costs—for a review, see [127]). Additionally, it would be interesting to analyze external barriers, since operational and managerial barriers are internal obstacles. Thus, further research is needed to explore the effect of each of the obstacles (e.g., lack of skills, lack of willingness among the management, lack of technology infrastructure, and lack of financial resources, among others) as well as the implementation strategies of working conditions (promoting and improving diversity and equality in the workplace, pro-environmental initiatives, etc.) to obtain helpful information for companies regarding which factors are most influential. In addition, future research should investigate the moderating effect of operational barriers in different contexts on the link between the adoption of environmental practices and sustainable product innovation.

## Figures and Tables

**Table 1:** Sample distribution (N = 3250 manufacturing firms).

	N	%
**Size**		
1–9 employees	1304	40.1
10–49 employees	906	27.9
50–249 employees	745	22.9
250 employees or more	267	8.2
**Country**		
*EU countries*	*2482*	*76.36*
Belgium	84	2.6
Bulgaria	106	3.3
Czechia	96	3
Denmark	78	2.4
Germany	76	2.3
Estonia	109	3.4
Greece	117	3.6
Spain	146	4.5
France	108	3.3
Croatia	138	4.2
Ireland	34	1
Italy	165	5.1
Republic of Cyprus	36	1.1
Lithuania	68	2.1
Latvia	79	2.4
Luxembourg	25	0.8
Hungary	127	3.9
Malta	25	0.8
The Netherlands	59	1.8
Austria	90	2.8
Poland	104	3.2
Portugal	97	3
Romania	104	3.2
Slovenia	132	4.1
Slovakia	111	3.4
Finland	93	2.9
Sweden	75	2.3
*Non-EU countries*	*768*	*23.64*
Bosnia and Herzegovina	45	1.4
Brazil	107	3.3
Canada	86	2.6
Iceland	44	1.4
Japan	70	2.2
Kosovo	42	1.3
Norway	37	1.1
North Macedonia	47	1.4
Serbia	56	1.7
Turkey	81	2.5
United Kingdom	53	1.6
United States of America	100	3.1

**Table 2:** Item loadings, average variance extracted (AVE), and composite reliability (CR) assessment.

Construct	Indicators	CR	Loadings	AVE
Firm growth	FG1	0.823	0.907	0.701
	FG2		0.761	
The adoption of environmental practices	AEP1	0.802	0.663	0.577
	AEP2		0.829	
	AEP3		0.778	
Managerial barriers	MB1	0.797	0.761	0.663
	MB2		0.864	
Operational barriers	OB1	0.718	0.937	0.579
	OB2		0.528	
Improvement in labor conditions	LC1	0.823	0.834	0.701
	LC2		0.896	
Sustainable product innovation	SPI1	0.781	0.732	0.642
	SPI2		0.864	

**Table 3:** Heterotrait–monotrait ratio (HTMT).

Construct	1	2	3	4	5	6	7	8
1. Firm growth								
2. The adoption of environmental practices	0.113							
3. Managerial barriers	0.057	0.224						
4. Operational barriers	0.130	0.256	0.570					
5. Improvement in labor conditions	0.161	0.741	0.296	0.282				
6. Sustainable product innovation	0.191	0.826	0.112	0.211	0.702			
7. Firm age	0.070	0.017	0.031	0.054	0.021	0.047		
8. Firm size	0.028	0.025	0.051	0.035	0.016	0.084	0.035	
9. Turnover	0.206	0.278	0.027	0.165	0.265	0.319	0.018	0.139

**Table 4:** Results of the structural model.

Construct	R^2^	R^2^ Adjusted	Q^2^	VIF
Firm age	-	-	1	1.006
Firm size	-	-	1	1.023
Turnover	-	-	1	1.19
Firm growth	-	-	0.160	1.038
Managerial barriers	-	-	0.077	1.084
Operational barriers	-	-	0.013	1.082
Improvement in labor conditions	-	-	0.249	1.364
The adoption of environmental practices	0.155	0.154	0.184	1.345
Sustainable product innovation	0.286	0.284	0.028	1.038

**Table 5:** Hypothesis testing.

Hypothesis	Relationship	Path	*t*-Value	*p*-Value	Conclusion
Direct effects					
H1	FG->SPI	0.036 **	2.405	0.008	Supported
Indirect effects					
H2	FG->AEP->SPI	0.028 ***	4.353	0.000	Supported
Interaction effects					
H3	AEP *MB->SPI	−0.038 *	2.317	0.010	Supported
H4	AEP *OP->SPI	0.001	0.083	0.467	Not significant
H5	AEP *ILC->SPI	0.145 ***	8.617	0.000	Supported
Control variables					
	Firm age->SPI	0.022	1.543	0.061	Not significant
	Firm Size->SPI	0.034 *	2.211	0.014	Significant
	Turnover->SPI	0.074 ***	4.617	0.000	Significant

* *p* < 0.05; ** *p* < 0.01; *** *p* < 0.001. Note: FG = firm growth; SPI = sustainable product innovation; AEM = the adoption of environmental practices; MB = managerial barriers; OP = operational barriers; ILC = improvement in labor conditions.

## Data Availability

The data presented in this study are openly available in GESIS Data Archive at doi.org/10.4232/1.13639, ZA7637.

## Appendix A

**Table A1:** Variable description.

	Construct	Item	Measure	Type
Independent variables	Firm growth	FG1	1 if the firm has grown in terms of turnover since 2016, 0 otherwise	Dichotomous
		FG2	1 if the firm has grown in terms of number of employees since 2016, 0 otherwise	Dichotomous
	The adoption of environmental practices	AEP1	Recycling or reusing materials	Dichotomous
		AEP2	Reducing consumption of or impact on natural resources (e.g., saving water or switching to sustainable resources)	Dichotomous
		AEP3	Saving energy or switching to sustainable energy sources	Dichotomous
	Managerial barriers	MB1	Lack of skills, including managerial skills	Dichotomous
		MB2	Lack of willingness among management staff	Dichotomous
	Operational barriers	OB1	Lack of technology infrastructure	Dichotomous
		OB2	Lack of financial resources, including those from available support schemes	Dichotomous
	Improvement in labor conditions	LC1	Improving the working conditions of employees	Dichotomous
		LC2	Promoting and improving diversity and equality in the workplace	Dichotomous
Dependent variable	Sustainable product innovation	SPI1	The introduction of innovation with environmental benefits, including innovations with an energy or resource efficiency benefit during the past 12 months	Dichotomous
		SPI2	The firm is actively developing sustainable products or services	Dichotomous
Control variables	Firm age		Number of employees	Continuous
	Firm size		The duration from the year in which the firm was established to the sample year (2019)	Continuous
	Annual turnover		It is represented by eight categories: (1) 150,000 or less	Categorical
			(2) more than 150,000 and up to 760,000	
			(3) more than 760,000 and up to 1.5 million	
			(4) more than 1.5 million and up to 3 million (5) more than 3 million and up to 7.6 million (6) more than 7.6 million and up to 15 million (7) more than 15 million and up to 76 million (8) more than 76 million	
